# Adenosine Metabotropic Receptors in Chronic Pain Management

**DOI:** 10.3389/fphar.2021.651038

**Published:** 2021-04-16

**Authors:** Livio Luongo, Francesca Guida, Sabatino Maione, Kenneth A. Jacobson, Daniela Salvemini

**Affiliations:** ^1^ Division of Pharmacology, Department of Experimental Medicine, Università della Campania “L. Vanvitelli”, Caserta, Italy; ^2^ IRCSS, Neuromed, Pozzilli, Italy; ^3^ Laboratory of Bioorganic Chemistry, NIDDK, National Institutes of Health, Bethesda, MD, United States; ^4^ Department of Pharmacology and Physiology, Saint Louis University School of Medicine, St. Louis, MO, United States

**Keywords:** metabotropic adenosine receptors, neuropathic pain, new targets, A1AR, A2AR, A3AR

## Introduction

The pharmacological treatment of chronic pain is still unsatisfactory. The cellular and molecular mechanisms at the basis of pain chronification are still poorly understood.

The commercially available drugs for treating chronic pain, including neuropathic pain, are effective in few patients and own several side effects that often limit the compliance of the patients. In addition, the opioids, which are the most potent analgesics, often fail in chronic pain conditions, especially in neuropathic pain syndromes. Moreover, it is well known that opiates can lead to addiction, tolerance and hyperalgesia. Therefore, new targets for treating neuropathic pain are needed. Purines, including ATP, ADP and adenosine and their receptors are deeply involved in the pathophysiology of neuropathic pain, particularly in the immune-mediated reactions that are responsible for the induction of the tactile allodynia, that represents the main symptom of neuropathic pain. The first response to the insult is mediated by the production of ATP and the activation of P2X receptors (Jacobson et al., 2020). In particular, the P2X4 receptors on microglia have been identified to be essential for the involvement of microglia cells in the pain pathophysiology (Trang et al., 2012). Beside the P2X, the P2Y receptors tend to boost the immune cells in response to nucleotides, with their ligands acting as immediate danger signals (Cekic and Linden, 2016). The subsequent stimulation of the metabotropic P1 adenosine receptors (ARs), of which are known four subtypes (A_1_, A_2A_, A_2B_, and A_3_), is overall associated with the reduction of both immunoinflammatory response and pain (Vincenzi et al., 2020).

In the present opinion paper we will focus on the role of the P1 adenosine receptors in the pathophysiology of chronic neuropathic pain. The involvement of P2X and P2Y receptors in chronic pain has been discussed elsewhere (Jacobson et al., 2020).

## Subsections Relevant for the Subject

### A_1_ Adenosine Receptor in Chronic Pain

The role of the A_1_AR in pain and nociception has been well described in both preclinical and clinical studies. Preclinical evidence showed a potent beneficial effect of several A_1_AR agonists in different animal models of chronic pain (Sowa et al., 2010; Luongo et al., 2012; Kan et al., 2018). It has been suggested that A_1_AR stimulation in peripheral nerves, might represents the molecular mechanism through which acupuncture exerts an antinociceptive effect (Goldman et al., 2010). A_1_AR knock out (KO) mice were used to evaluate the role of the A_1_AR in nociception. Under normal conditions, as well as during inflammatory or neuropathic pain, A_1_AR KO animals showed a lower thermal threshold as compared to the wild-type (WT) mice. A_1_AR KO mice also showed a reduced antinociceptive response to morphine given intrathecally, but not systemically (Wu et al., 2005). This is in agreement with other data showing that intrathecal morphine generates an antiallodynic effect through A_1_AR activation (Zhang et al., 2005). A_1_AR is widely expressed in the nervous system. A_1_AR activation induces presynaptic inhibition of primary afferent fibers at the dorsal horn level. This inhibition is associated with decreased release of glutamate, substance P, and other proinflammatory mediators from primary afferents fibers to the spinal cord. A_1_AR also hyperpolarizes dorsal horn neurons by increasing K^+^ conductance and reducing Ca^2+^ influx (Bai et al., 2017).

Moreover, recent evidence also highlights the expression of the A_1_AR in primary microglia cell cultures (Luongo et al., 2014), assuming its potential role also in neuroinflammatory processes at the basis of the induction of tactile allodynia. Clinical studies also have been carried out for A_1_AR agonists. However, several side effects, especially at the cardiovascular level were associated to the use of those compounds (Zylka, 2011).

### A_2A_ and A_2B_ Adenosine Receptors in Chronic Pain

The role of the A_2A_AR in pain is still a matter of debate since both pronociceptive and antinociceptive effects have been reported in animal models of inflammatory and neuropathic pain. Several reports highlighted the long lasting antiallodynic effect of the spinally-injected A_2A_AR agonists (Loram et al., 2009; Kwilasz et al., 2018). CGS21680, a selective A_2A_AR agonist, reduced the formalin-induced nocifensive behavior in both the early (0–15°minutes) and late (15–60°minutes) phases in a mouse model of formalin-induced inflammatory pain (Nalepa et al., 2010).

On the other hand, several papers showed a facilitative role of the A_2A_AR on nociceptive threshold. In particular, it has been suggested that mice lacking the A_2A_AR are less responsive to the noxious stimuli and, in these mice, the spinal cord neurons seems to be less active (Hussey et al., 2007; Hussey et al., 2010). Moreover, A_2A_AR KO mice showed reduced tactile allodynia as compared to the wild type animals in a mouse model of neuropathic pain due to the sciatic nerve injury (Bura et al., 2008).

### A_3_ Adenosine Receptor in Chronic Pain

The role of the A_3_AR emerged only recently in the pain field. The analgesic effect of adenosine was, in fact, believed to be mediated mainly by A_1_AR stimulation and, at least in part, by A_2A_AR (Sawynok, 2016; Kwilasz et al., 2018). In addition, A_1_AR and A_2A_AR modulating drugs, although effective in the preclinical models, did not reach clinical experimentation for their important cardiovascular side effect (Zylka, 2011; Jacobson et al., 2020). Contrary to what emerged from the early studies showing A_3_AR levels in the brain that were difficult to detect (Rivkees et al., 2000), the A_3_AR is expressed in different areas of the central nervous system (CNS) of both rodents and humans (Yaar et al., 2002; Jacobson et al., 2018). Besides the A_3_AR expression in neurons, which is lower as compared to the A_1_AR and A_2A_AR in physiological conditions, there is a high expression of this receptor in immune cells in the periphery and CNS. Indeed, it has been demonstrated that A_3_AR is expressed by astrocytes, oligodendrocytes, microglia, and endothelial cells, including in human tissue (Zhang et al., 2016). This is important since recent evidence highlighted the key role of the microglia and astrocytes in the induction and maintenance of tactile allodynia, which represents a major symptom associated with neuropathic pain.

After early confusing reports about the involvement of the A_3_AR in chronic pain, recent evidence supports a pivotal role of this receptor in the reduction of tactile allodynia in different preclinical models of neuropathic pain. The recent synthesis of highly selective agonists for A_3_AR (>10,000-fold in comparison to other AR subtypes) and the possibility to use A_3_AR KO animals paved the way for more definitively investigating these receptors in the pain axis. In fact, the selective pharmacological stimulation of the A_3_AR induced pronounced and prolonged antiallodynic effects in traumatic nerve-injury, chemotherapy-induced and other models of neuropathic pain (Bar-Yehuda et al., 2011; Chen et al., 2012; Fishman et al., 2012; Janes et al., 2015; Little et al., 2015; Tosh et al., 2015; Terayama et al., 2018; Wahlman et al., 2018; Stockstill et al., 2020). Moreover, A_3_AR-selective agonists reduced the formalin-induced nocifensive behaviour and diabetic neuropathy (Yan et al., 2016; Petrelli et al., 2017). The A_3_AR seems to exert its beneficial effect at different levels of the pain axis. Indeed, the receptors can be activated at the spinal cord (SC) and rostral ventromedial medulla (RVM) levels, where the mRNA has been found (Little et al., 2015). Moreover, the selective activation of the A_3_AR can recruit several downstream pathways (Cohen and Fishman, 2019; Kim et al., 2019). These pleiotropic mechanisms might be the reason for the high efficacy of the A_3_AR agonists. Among several downstream mechanisms, the A_3_AR stimulation has been shown capable of reducing the expression of proinflammatory cytokines including tumor necrosis factor (TNFα) and interleukin-1β (IL-1β), and enhancing the expression of antiinflammatory cytokines, such as interleukin-10 (IL-10) and interleukin-4 (IL-4) in the SC (Janes et al., 2015; Wahlman et al., 2018). Intriguingly, it has also been observed that the A_3_AR signaling is associated with a spinal mechanism of action that modulates the chloride potassium symporter 5 (KCC2 transporter), which has been shown to be downregulated and, in turn, responsible for the GABAergic gradient shift from inhibitory to excitatory signaling in neuropathic pain (Ford et al., 2015). This latter mechanisms is very important for the establishment of tactile allodynia, which also involves microglia cells (Coull et al., 2005). Coppi and coworkers also highlighted the capability of A_3_AR agonists to inhibit the pronociceptive N-type Ca^2+^ currents and cell excitability in dorsal root ganglion (DRG) neurons (Coppi et al., 2019). This latter mechanism has also been suggested by Lucarini and colleagues in a model of visceral pain in rats (Lucarini et al., 2020).

**FIGURE 1:**
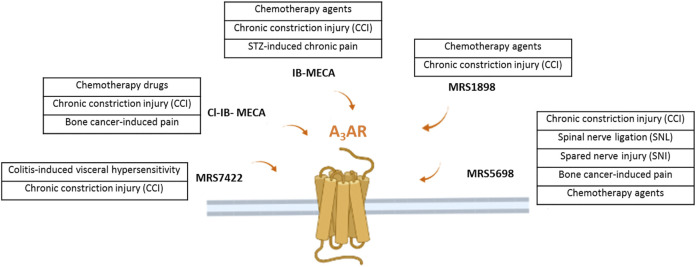
The figure illustrates the several A3AR agonists use in different preclinical model of neuropathic pain of various origins.

Very recently, it has been demonstrated that the morphine-induced tolerance could also be mediated by the A_3_AR, suggesting a possible use of the A_3_AR agonists as adjuvant therapy in combination with opioids (Doyle et al., 2020). Coadministration of an A_3_AR agonist at a low dose also reduced withdrawal behavior following morphine administration in rats, without reducing morphine’s antinociceptive effect. A scheme summarizing the molecules acting on the A3AR in different preclinical models of neuropathic pain is shown in Figure 1.

## Discussion

Purinergic signaling is involved in pain transmission. Data from different laboratories suggested that adenosine metabotropic receptors play key role in chronic pain models. In the previous years, the focus on adenosine in pain was mostly directed to the A_1_ and A_2A_ receptors and their pharmacological manipulation. Cardiovascular side effects are the most limiting for the use of these compounds clinically.

More recent research has revealed the central role of the A_3_AR and its pharmacological manipulation for chronic pain of various origins. The efficacy of the A_3_AR agonists could be due to their pleiotropic mechanism of action, without exerting pronounced cardiovascular side effects. Interestingly, the antinociceptive effect of the A_3_AR pharmacological stimulation is independent from the recruitment of the opioid or cannabinoid systems, it does not alter physiological pain, thus, avoiding the problems related to the tolerance and abuse (Janes et al., 2016). Worthy of note is also the capability of A_3_AR to synergize with other drugs commonly used for treating chronic pain including opioids, amitriptyline and gabapentin (Chen et al., 2012).

To conclude, it is clear that the purines deserve further research attention in the pain field offering very promising results indicative of future potential. In particular, A_3_AR seems to represent a promising candidate for developing safer and more effective drug treatment for neuropathic pain.
